# Subsets of Tissue CD4 T Cells Display Different Susceptibilities to HIV Infection and Death: Analysis by CyTOF and Single Cell RNA-seq

**DOI:** 10.3389/fimmu.2022.883420

**Published:** 2022-06-16

**Authors:** Xiaoyu Luo, Julie Frouard, Gang Zhang, Jason Neidleman, Guorui Xie, Emma Sheedy, Nadia R. Roan, Warner C. Greene

**Affiliations:** ^1^ Gladstone Institute of Virology, San Francisco, CA, United States; ^2^ Department of Urology, University of California, San Francisco, San Francisco, CA, United States; ^3^ Department of Medicine, University of California, San Francisco, San Francisco, CA, United States; ^4^ Department of Microbiology & Immunology, University of California, San Francisco, San Francisco, CA, United States

**Keywords:** human immunodeficiency virus (HIV), cell death, apoptosis, pyroptosis, lymphoid tissues, mass cytometry, flow cytometry, single-cell RNA-seq

## Abstract

CD4 T lymphocytes belong to diverse cellular subsets whose sensitivity or resistance to HIV-associated killing remains to be defined. Working with lymphoid cells from human tonsils, we characterized the HIV-associated depletion of various CD4 T cell subsets using mass cytometry and single-cell RNA-seq. CD4 T cell subsets preferentially killed by HIV are phenotypically distinct from those resistant to HIV-associated cell death, in a manner not fully accounted for by their susceptibility to productive infection. Preferentially-killed subsets express CXCR5 and CXCR4 while preferentially-infected subsets exhibit an activated and exhausted effector memory cell phenotype. Single-cell RNA-seq analysis reveals that the subsets of preferentially-killed cells express genes favoring abortive infection and pyroptosis. These studies emphasize a complex interplay between HIV and distinct tissue-based CD4 T cell subsets, and the important contribution of abortive infection and inflammatory programmed cell death to the overall depletion of CD4 T cells that accompanies untreated HIV infection.

## Introduction

Massive depletion of CD4 T cells by HIV is a hallmark of untreated HIV infection (1–6). The pernicious depletion of these cells leads to AIDS, characterized by frequent opportunistic infections, emergence of various cancers, and death (6). Despite decades of study, the underlying mechanism of CD4 T cell depletion during HIV infection remains incompletely understood.

Multiple mechanisms have been reported to contribute to HIV-associated CD4 T cell depletion (7). These include autophagy of productively infected CD4 T cells, viral protein induced apoptosis (e.g. Env, Tat, Nef), and activation-induced cell death (8–13). These mechanisms were mainly demonstrated in blood-derived CD4 T cells experimentally infected with HIV. However *in vivo*, HIV-associated cell death predominantly occurs in lymphoid tissues (14–16). Our group has identified abortive infection and pyroptotic programmed cell death as a major driver of the cell death occurring in lymphoid tissue-derived but not blood-derived CD4 T cells (17, 18). Abortive infection and pyroptotic cell death affect non-permissive CD4 T cells, also called “bystander” T cells, which die from HIV intrusion even though they do not sustain a productive infection. Pyroptosis is a highly inflammatory form of programmed cell death characterized by gasdermin D-induced pore formation in the plasma membrane followed by cellular swelling and rupture (19–21). This pyroptotic death was observed during *ex vivo* HIV infection of human lymphoid aggregated cultures (HLAC) formed with either human tonsil or spleen tissue (22). The signaling pathway leading to activation of this death pathway entails HIV entry into non-permissive bystander CD4 T cells, followed by stalling of the infective process during reverse transcription. The ensuing accumulation of viral DNAs is detected by the IFI16 sensor, which triggers inflammasome formation and cell death by caspase 1-mediated pyroptosis. Abortive infection followed by pyroptotic cell death is the fate of the majority of HLAC CD4 T cells. By comparison, only a small portion (~ 5%) of the CD4 T cells in HLAC are able to support productive infection by HIV (18, 23–25), However, these productively infected cells provide the source of virus driving abortive infection of bystander cells, which depends on cell-to-cell viral transmission (24). Ultimately the productively infected cells die chiefly by caspase 3-mediated apoptosis (18).

While these prior studies have revealed distinct mechanisms underlying HIV-associated CD4 T cell death in lymphoid tissues, certain details of the process remain unclear. In particular, it is unknown whether specific CD4 T cell subsets are preferentially depleted over others, and if so, what determines the different fates of these cells. Although preferential killing of memory versus naïve CD4 T has been reported (26), these studies involved blood-derived cells. No studies to date have examined whether HIV kills different CD4 subsets within lymphoid tissues. Furthermore, T cells subsets are far more complex than just memory and naïve subsets (27). Indeed, CD4 T cells are highly heterogeneous (27, 28), and with recent developments in high-parameter cellular phenotyping including mass cytometry (CyTOF), a more complete view of the diversity of CD4 T cell subsets has emerged (29). CyTOF, which involves the use of antibodies coupled to lanthanide metals instead of the fluorophores used in flow cytometry (FACS), enables simultaneous quantitation of ~ 40 different protein parameters uncompromised by spectral overlap (30). Recently, CyTOF has been used to interrogate the cellular subsets of CD4 T cells preferentially susceptible to productive infection by HIV (31–34).

CyTOF-mediated high-parameter phenotyping also enables the implementation of the Predicted Precursor as determined by SLIDE (PP-SLIDE), a bioinformatics approach that predicts the original state of cells prior to the remodeling that HIV infection induces (34). This viral remodeling, which causes the up- or down-regulation of various cellular proteins, is a prominent feature of HIV infection (35, 36). Viral remodeling raises a problem when trying to subset HIV-susceptible cells, because it may alter expression levels of the antigen used to define a subset. However, one can overcome this problem by simultaneous analysis of many antigens, since the collective information attained in this manner is sufficient to capture the subset identity of the original cell targeted by the virus. In this manner, the analysis of CyTOF datasets using PP-SLIDE allows prediction of the original phenotypes of preferentially-infected T cell subsets, and such predictions have been validated in multiple systems including within HIV-infected tonsil cells (31–34). To date, however, CyTOF and PP-SLIDE have not been implemented to understand HIV-associated cell death in subsets of human CD4 T cells present in lymphoid tissue.

In the current study, we combined the HLAC model of HIV-associated cell death in lymphoid tissues with high-dimensional single-cell phenotyping by CyTOF paired with PP-SLIDE analysis, to better understand the mechanisms underlying HIV-associated T cell depletion occurring in the CD4 T cell subsets residing in lymphoid tissue. We find that most cell death does not occur among productively-infected cells but rather among multiple subsets of bystander CD4 T cells in the infected cultures. We further identify specific surface markers of the subsets preferentially lost as bystanders and interrogate these subsets using single-cell transcriptomics to assess their mechanism of cell death.

## Results

### HIV Differentially Depletes CD4 T Cell Subsets in the HLAC System

**Figure 1:**
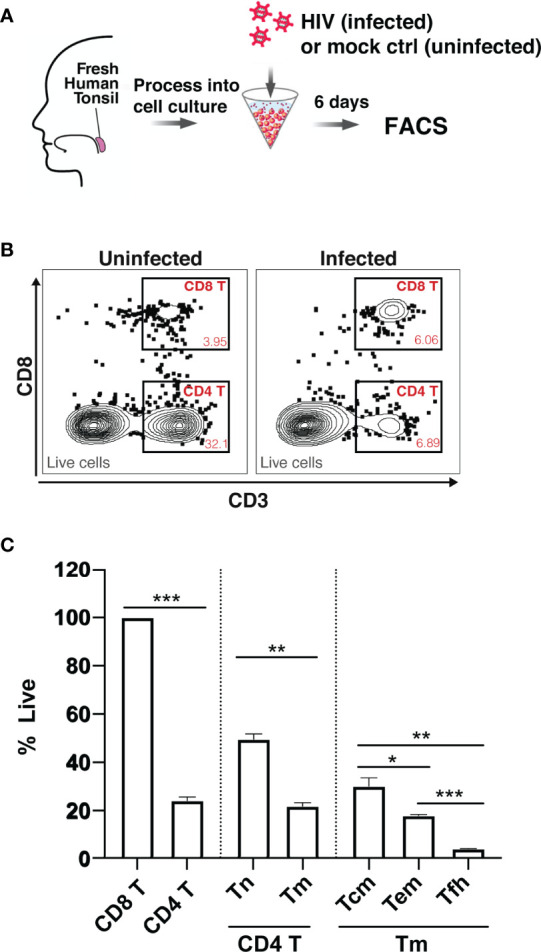
Preferential death of CD4 T cells in tonsil HLAC specimens infected *ex vivo* with HIV. **(A)** Schematic of the HLAC collection and *ex vivo* infection. Fresh human tonsil cells were mock-treated or infected with HIV.GFP by spinoculation. Six days later samples were harvested for analysis by FACS. **(B)** Gating to identify CD8 T (CD8+CD3+), CD4 T (CD8-CD3+) in infected cultures (right) and uninfected control culture (left). Preceding parent gates are indicated at the lower left corner. Numbers correspond to percentages of cells in the indicated gate. Data for this one donor is representative of the 6 donors. **(C)** Quantification of CD4 T Naive (Tn), CD4 T memory (Tm), and CD4 T memory subsets including central memory (Tcm), effector memory (Tem), and T follicular helper (Tfh) cells, as identified by sequential gating (see **Figure S1**). For each subset, the percentage of live cells relative to uninfected control (% live) is shown. Data were normalized to CD8 T cell counts (details described in Materials and Methods). The data represent mean +SD of an experiment performed in triplicate. These data represent 6 donors studied in 3 independent experiments. *p ≤ 0.05; **p ≤ 0.01; ***p ≤ 0.001; no label: not significant, *p > 0.05.* Significance was measured by paired Student’s T test.

To assess whether HIV differentially depletes different subsets of tissue CD4 T cells, we measured HIV-associated cell depletion in several discrete T cell subsets using flow cytometry first (**Table S1**). Fresh HLAC were prepared as previously described (22), either mock infected or infected with an X4-tropic HIV.GFP reporter virus, and cultured for 6 days before analysis (**Figure 1A**). As previously reported, we observed a marked loss of CD4 T cells (defined as CD3+CD8-) in the infected culture (frequency of 6.89%) as compared to the uninfected control (frequency of 32.1%) (**Figure 1B**). In contrast and as expected, CD8 T cell numbers (defined as CD3+CD8+) did not decrease in infected cultures. Normalization of the leftover live CD4 T cell counts in the infected culture to the CD8 T cell counts (details described in Materials and Methods) confirmed significant depletion of the CD4 T cells (**Figure 1C**). Interestingly, HIV-associated CD4 T cell depletion was more pronounced in the CD4 T memory (Tm) relative to in the CD4 T naïve (Tn) cells. Furthermore, within the memory compartment, T follicular helper (Tfh) cells were preferentially killed over either effector memory (Tem) or central memory (Tcm) cells (**Figures 1C**; **S1**). These FACS results suggest that while HIV infection is associated with T cell depletion by HIV in multiple CD4 T subsets, and that the levels of depletion differ within these subsets.

### Implementation of CyTOF and PP-SLIDE for Deep Phenotyping of the HIV-Depleted HLAC Cells

**Figure 2:**
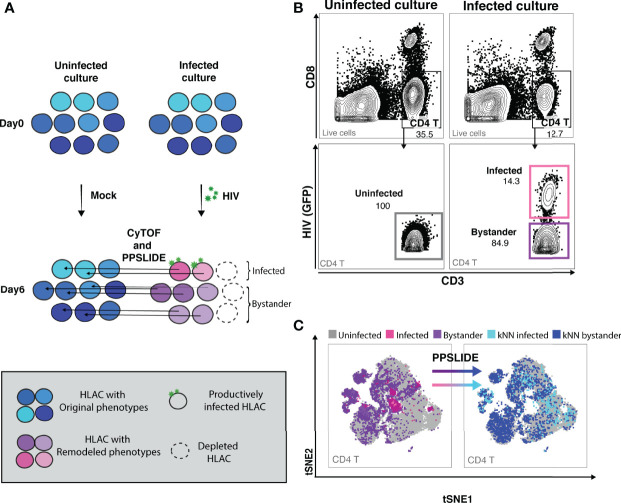
CyTOF and PP-SLIDE analysis of HIV-associated killing in HLAC system. **(A)** Schematic of CyTOF and PP-SLIDE experimental strategy. HLAC cells were mock-treated or infected with HIV.GFP for 6 days and then processed for CyTOF analysis. Only cells that are productively infected express GFP. For every infected cell (in pink), we employed PP-SLIDE to trace it back to the most phenotypically similar cell in the uninfected culture using a k-nearest neighbor (kNN) approach. This kNN infected cell harbors the predicted phenotype of the infected cell prior to HIV-induced cell remodeling. Similarly, bystander cells in the HIV-exposed culture (purple) were also mapped to their predicted state prior to infection using PP-SLIDE. The key is shown in the grey inset. **(B)** Example of manual gating strategies to identify the following CD4 T subsets: uninfected (CD3+CD8-, uninfected culture, gray gate), infected (CD3+CD8-HIV+, infected culture, pink gate) and bystander (CD3+CD8-HIV-, infected culture, purple gate). Preceding parent gates are indicated at the lower left corner. Numbers correspond to percentages for each gate. **(C)** tSNE plots showing infected (pink dots) and bystander (purple dots) CD4 T cells overlayed onto uninfected CD4 T cells (gray dots). Both infected and bystander CD4 T cells were remodeled as suggested by their presence in regions of the tSNE not occupied by uninfected CD4 T cells. Using PP-SLIDE, infected and bystander CD4 T cells were converted to their predicted original states, kNN infected (aqua dots) and kNN bystander (blue dots) respectively. Preceding parent gates are indicated at the lower left corner.

To study HIV-associated depletion in greater depth, and to take advantage of the PP-SLIDE bioinformatics approach that corrects for virus-induced remodeling of cellular phenotypes, we designed a new 38-parameter CyTOF panel able to distinguish a wide range of T cell phenotypic subsets (**Table S2**). Even if some parameters are altered by infection, the high number of CyTOF parameters allows efficient backtracking to the original cell population *via* PP-SLIDE. As recently described (34), PP-SLIDE uses the 40 CyTOF parameters to match each cell in the infected culture to its “k-nearest neighbor” (kNN) cell in the uninfected control population (**Figure 2A**, and Materials and Methods). HIV infection is known to remodel productively-infected cells (“infected”), but non-productively-infected (“bystander”) cells could also be remodeled by the inflammatory environment of infected cells. To examine killing, we infected HLAC cells with HIV-GFP for 6 days followed by CyTOF and PP-SLIDE analysis. Using CD4 T cell markers and HIV reporter (GFP) expression, we selected infected (GFP+) and bystander (GFP-) CD4 T cells in the infected HLAC population, as well as the CD4 T cells present in the uninfected control (**Figures 2B**; **S3**). We then visualized the phenotypes of CD4 T cells using t-distributed stochastic neighbor embedding (tSNE), a dimension reduction visualization method (37) (**Figure 2C**). Remodeling of both infected (pink dots) and bystander (purple dots) CD4 T cells from the infected culture was suggested by the fact that many of these cells did not map to regions of the tSNE plot occupied by CD4 T cells from the uninfected sample (gray dots) (**Figure 2C**, left panels). In contrast, after application of PP-SLIDE, their “nearest neighbors” (“kNN infected” and “kNN bystander”) localized within the regions occupied by the uninfected CD4 T cells in the tSNE plot (**Figure 2C**, right panels, aqua, blue and gray dots). As the kNN infected and kNN bystander cells harbor the predicted features of the original infected and bystander CD4 T cells, prior to remodeling, for the remainder of the study we simply refer to these cells as “infected” and “bystander” cells.

### Tissue Memory CD4 T Cells Are Preferentially Killed and Infected Compared to Naïve CD4 T Cells

**Figure 3:**
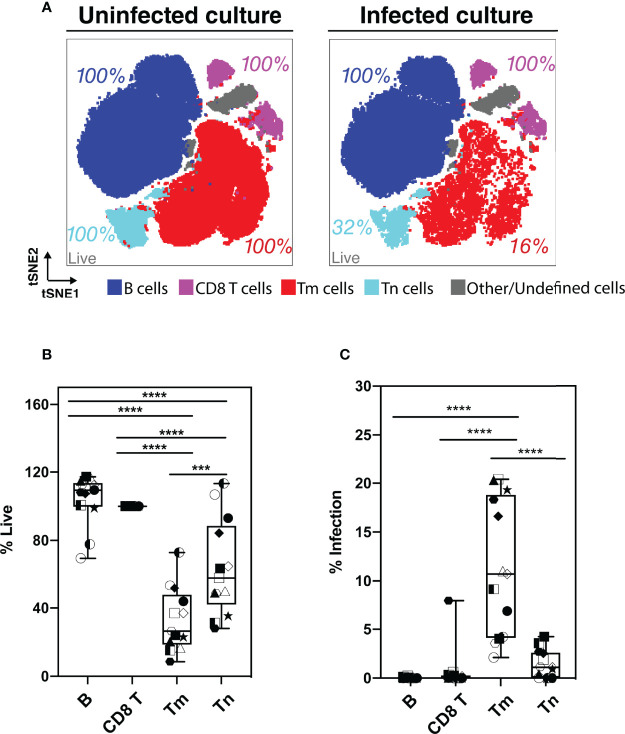
Assessment of preferential killing and infection of main subsets of HLAC cells (B, CD8T, CD4 T memory, and CD4 T naïve cells). **(A)** tSNE plots of total HLAC cells from uninfected culture (left panel), and HIV infected culture (right panel). HLAC cells were classified and colored by main subsets including B (CD19+) (dark blue), CD8 T (CD3+CD8+) (purple), Tm (CD3+CD4+CD45RO+CD45RA-) (red), Tn (CD3+CD4+CD45RO-CD45RA+) (light blue), and Other/Undefined (CD19-CD3-, gray). These subsets were defined using FlowSOM and cell surface marker expression (**Figure S4**). Live cell percentage (% live) in each subset were calculated as described in the Materials and Methods, and are labeled beside each cell subset. Preceding parent gates are indicated at the lower left corner. **(B, C)** Proportions of live **(B)** and infected **(C)** cells among B, CD8 T, Tm, and Tn cells from 6 donors, calculated as described in Materials and Methods. Symbols represents technical repeats from 6 donors. Each donor is represented as a shape with technical repeats represented with different type of fill (details described in Materials and Methods). ***p ≤ 0.001; ****p ≤ 0.0001; no label: not significant, p > 0.05. Significance was measured by one-way ANOVA with repeated measurements followed by *post-hoc* tests (n = total paired-wise comparisons). Paired effect between Tm and Tn is shown by estimation plots in **Figure S8**.

Although our flow cytometry data suggested that HIV-associated killing occurred preferentially among memory CD4 T cells (Tm cells), this might have been an artifact due to the remodeling of the surviving bystander cells into naïve-like CD4 T cells (Tn cells). To address this possibility, we classified the HLAC cells into the main immune subsets (B, CD8 T, Tn, and Tm cells) by applying the FlowSOM clustering approach (38) to the PP-SLIDE corrected CyTOF data (**Figures 3A**; **S4**). We found that both Tm and Tn cells were killed by HIV but at different levels. Compared to the uninfected culture, the infected culture had lost 84% of its Tm cells and 68% of its Tn cells (**Figure 3A**, red and aqua). The higher loss of Tm over Tn cells was statistically significant (n=6 donors, **Figure 3B**), confirming preferential depletion of memory over naïve CD4 T cells.

Preferential killing of the Tm subset could result from higher permissivity to productive infection leading to higher viral-induced cytotoxicity. To test if Tm cells were also preferentially infected by HIV, we measured the level of productive infection (% productively infected) in Tm and Tn cells (**Figure 3C**). We found a higher proportion of productively-infected cells among Tm than Tn cells; in fact, the majority of the Tn cells (> 95%) were resistant to HIV infection. Our observation that Tn cells are depleted but poorly infected suggests that many Tn cells die as bystander cells.

### Preferentially-Killed and Preferentially-Infected Tm Subsets Do Not Fully Overlap

**Figure 4:**
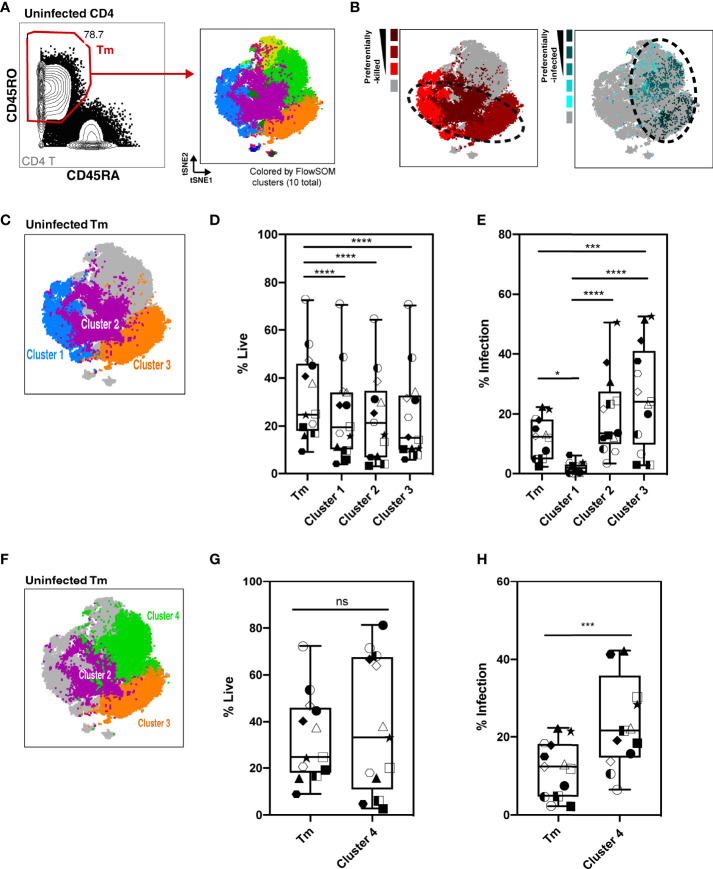
Characterization of HIV-associated killing and infection in FlowSOM-defined Tm subsets. **(A)** Visualization of HLAC Tm cell clusters by FlowSOM, an algorithm based on a self-organizing map. *Left panel:* Example of Tm gating strategy (CD45RO+CD45RA-) (left). *Right panel:* Example of a tSNE plot colored by FlowSOM clusters (10 total). Preceding parent gates are indicated at the lower left corner. Numbers correspond to percentages for each gate. **(B)** *Left panel:* Preferentially-killed clusters colored in different shades of red (darker red represents a higher level of killing). *Right panel:* Preferentially-infected clusters colored in different shades of aqua (darker aqua represents a higher infection rate). Other clusters were colored in gray. Dotted circles on the tSNE plot shows that the preferentially-killed (left) and preferentially-infected (right) subsets do not completely overlap phenotypically. **(C)** Location of three preferentially-killed subsets shown on a tSNE plot: cluster 1 (blue), cluster 2 (purple), and cluster 3 (orange). Other clusters were colored as gray. **(D, E)** Proportion of live cells **(D)** and infection rate **(E)** of each of the 3 preferentially-killed subsets from 6 donors, plotted as box plots. Symbols represents technical repeats from 6 donors. Each donor is represented as a shape with technical repeats represented with different type of fill (details described in Materials and Methods). ***p ≤ 0.001; ****p ≤ 0.0001; no label: not significant, p > 0.05. Significance was measured by one-way ANOVA with repeated measurements followed by *post-hoc* tests for multiple comparisons correction. (n = total paired-wise comparisons). **(F)** Location of the three preferentially-infected subsets of Tm cells on the tSNE plot: cluster 2 (purple), cluster 3 (orange) and cluster 4 (green). Other Tm clusters are colored as gray. Tm (**G, H)** Proportion of live cells **(G)** and infection rate **(H)** in Tm and in cluster 4 from 6 donors, plotted as box plots. Symbols represents technical repeats from 6 donors. Each donor is represented as a shape with technical repeats represented with different type of fill (details described in Materials and Methods). ***p ≤ 0.001; n.s., not significant, p > 0.05. Significance was measured by paired Student’s T test. Paired effect between Tm and each of the 4 clusters was shown by estimation plots in **Figure S9**.

As Tm cells were preferentially killed compared to Tn cells, we focused the rest of our analysis on Tm cells. We characterized killing within different Tm subsets and investigated whether the higher HIV-associated killing of Tm subsets correlated with their permissivity to productive infection. First, the Tm cells were subjected to FlowSOM clustering (**Figure 4A**). Tm subsets that were preferentially killed (red) and -infected (aqua) were identified by their higher level of death or infection, respectively, as compared to those found in total Tm cells (details described in Materials and Methods, **Figure S5**). Interestingly, although there was overlap, cells preferentially killed resided in distinct areas of the tSNE relative to those preferentially infected, suggesting they represent different subsets (**Figure 4B**, dotted circles). Next, we looked into each of the preferentially-killed Tm subsets (clusters 1, 2, and 3), and quantitatively assessed their levels of HIV-associated depletion and infection (**Figures 4C–E**). Among these three preferentially-killed clusters, cluster 1 was preferentially depleted but was highly resistant to productive infection, suggesting that these cells likely died as bystander cells (**Figures 4D, E**). In contrast, clusters 2 and 3 were preferentially depleted and were highly permissive to productive infection, suggesting death in these two clusters might be attributed at least in part to apoptosis triggered by a productive infection. In addition to clusters 2 and 3, cluster 4 was also preferentially infected (**Figures 4E, F, H**). However, unlike cluster 2 and 3, cluster 4 was not preferentially killed (**Figure 4G**). Taken together, these findings suggest that Tm subsets are differentially depleted by HIV. Among the preferentially-killed Tm subsets, some are resistant to productive infection (cluster 1) while others are highly permissive to productive infection (cluster 2, 3). Moreover, high permissivity to infection is not always associated with high killing of Tm cells, as exemplified by cluster 4 (Table S3).

### Phenotypic Features Associated With Preferentially-Killed Subsets

**Figure 5:**
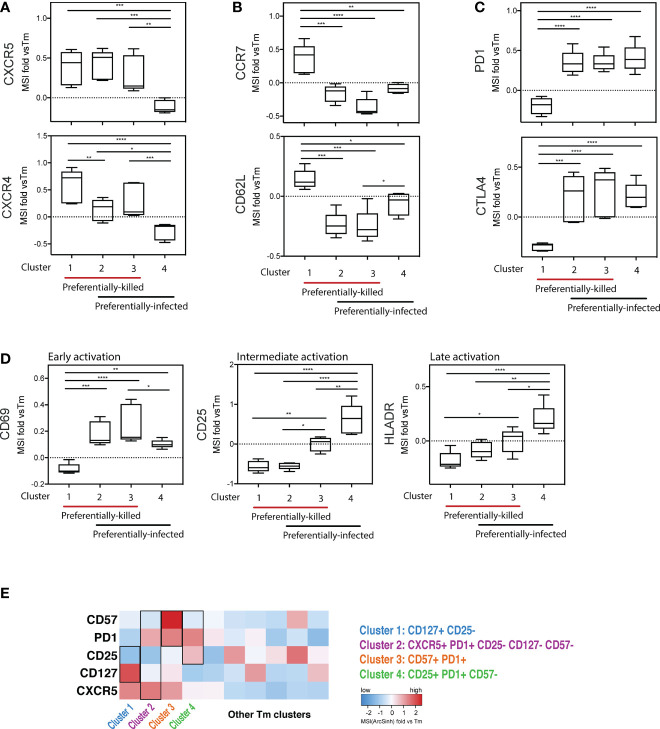
Deep phenotyping of the preferentially-killed versus preferentially-infected CD4 Tm subsets. **(A–D)** Expression levels of selected cell markers in clusters 1-4 (identified in **Figure 4**) relative to the CD4 Tm population (MSI fold vs Tm). For each marker, the mean expression intensity (MSI) of the cluster is compared to the MSI of the Tm population, according to the following equation: (MSI_Cluster_ / MSI_Tm_) -1. If this value is > 0, the cluster cells expressed a higher level of the selected cell marker on average than did the total Tm cells. On the x-axes, clusters 1-4 were grouped into 2 categories including preferentially-killed (red line) and preferentially-infected (black line). The plots combine data from 6 donors. *p ≤ 0.05; **p ≤ 0.01; ***p ≤ 0.001; ****p ≤ 0.0001; no label: not significant, p > 0.05. Significance was measured by one-way ANOVA with repeated measurements followed by *post-hoc* tests for multiple comparisons correction. (n = total paired-wise comparisons). **(E)** Heatmap of the 5 cell markers that can be used to distinguish cells belonging to clusters 1-4 from other Tm cells. The marker combinations corresponding to the 4 clusters is listed on the right. All MSI values used in **Figure 5** were arcsinh transformed.

To further characterize the phenotypic features of preferentially-killed and preferentially-infected Tm subsets, we assessed expression levels of various antigens within our CyTOF panel (**Figure 5**). We found that all preferentially-killed subsets (clusters 1, 2, and 3) expressed high levels of the two chemokine receptors CXCR5 and CXCR4 (**Figure 5A**). While CXCR5 defines Tfh-like cells, CXCR4 is the co-receptor used by our reporter virus, suggesting that efficient entry of HIV into cells may underlie preferential HIV-associated cell death. In contrast, unique features of the preferentially-infected subsets (clusters 2, 3, and 4) included low expression levels of CCR7 and CD62L, markers of Tcm cells (**Figure 5B**). These results suggest that Tm cells with a Tem phenotype (CCR7-CD62L-) (33) are preferentially infected. The preferentially-infected clusters also expressed high levels of the exhaustion markers PD1 and CTLA4 (**Figure 5C**). As these antigens are also markers of activated cells (39, 40), we assessed the activation status of the preferentially-infected subsets. We observed that the early activation marker CD69 was high on all the preferentially-infected clusters, while CD25 and HLADR, which are upregulated at later stages of T cell activation, were preferentially expressed only in cluster 4 (**Figure 5D**).

Interestingly, cluster 4 was the only subset that was preferentially infected but spared from HIV-associated killing. This suggests that preferentially-infected cells resistant to HIV-induced killing exhibit the following features: 1) low expression of the CXCR4 co-receptor, 2) a Tem phenotype (CCR7-CD62L-), and 3) high expression of activation markers (PD1, CTLA4, CD69, CD25, and HLADR) (**Figures 5A–D**). In contrast, among the four analyzed Tm clusters, cluster 1 was the only one preferentially susceptible to HIV-associated killing but not productive HIV infection, suggesting its propensity to be killed by bystander mechanisms (**Figures 4D, E**). The phenotypes associated with cluster 1 were: 1) high CXCR4 expression, and 2) a Tcm phenotype (CCR7+CD62L+), 3) low expression of exhaustion markers (PD1-CTLA4-), and 4) low expression of activation markers (CD69-CD25-HLADR-) (**Figures 5A–D**).

Further mining of the phenotyping data revealed that various combinations of CD127, CD25, PD1, CD57, and CXCR5 could be used to uniquely define the four subsets (**Figure S7**; **Table S3**). In particular, we found cluster 1, the only cluster preferentially killed by bystander mechanisms, could be defined as CD127+CD25- Tm cells. Cluster 4, which was highly permissive but not preferentially killed, could be defined as CD25+PD1+CD57- Tm cells. Clusters 2 and 3 were characterized by CD57+PD1+ Tm cells and CXCR5+PD1+CD25-CD57-CD127- Tm cells, respectively, and both of these subsets were both preferentially killed and infected. These identified markers enable characterization of preferentially killed/infected subsets without a need for 40 parameter clustering.

### Expression of Viral Restriction Factors and Cell Death Pathways Distinguish Tm Cells That Die as Bystander Cells From Tm Cells That Survive Productive Infection

**Figure 6:**
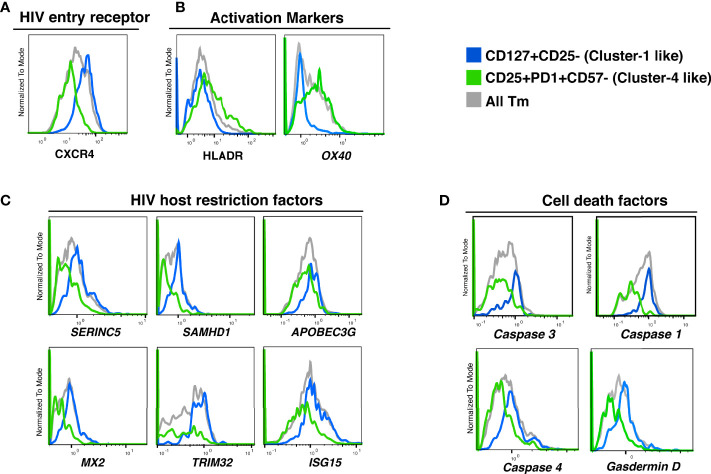
Proteins and transcripts distinguishing CD4 Tm cells preferentially killed as bystander cells from CD4 Tm cells able to survive productive infection. **(A–D)** Histograms of the expression of **(A)** HIV entry receptors, **(B)** activation markers, **(C)** HIV host restriction factors, and **(D)** cell death related factors in CD127+CD25- Tm (cluster 1-like, blue), CD25+PD1+CD57- Tm (cluster 4-like, green), and total Tm cells (grey). Names in italics indicate markers identified as transcripts. The data represented were collected from 2 donors in 2 independent experiments.

To refine our understanding of what distinguishes the different Tm cell subsets, we analyzed their transcriptional profiles. Taking advantage of our ability to identify preferentially killed/infected subsets using only a limited number of surface markers, we then implemented Antibody-Seq, which isolates cells with DNA oligo-barcoded antibodies directed as their surface antigens and subjects them to single-cell RNA-seq (41, 42). We focused this analysis on the two extreme categories: the preferentially-killed but not infected subset (cluster 1-like), which could be defined as CD127+CD25- Tm cells; and the preferentially-infected but not killed subset (cluster 4-like), which could be defined as CD25+PD1+CD57- Tm cells. Consistent with our CyTOF data (**Figure 5A**), the cluster 1-like cells expressed high levels of CXCR4 protein (**Figure 6A**). In contrast, the cluster 4-like cells expressed lower levels of CXCR4; this level is presumably sufficient to support productive infection but not viral-induced killing, as previously suggested (24). In addition, similar to our CyTOF result (**Figure 5D**, right), we found cluster 1-like cells expressed lower levels of HLADR, while cluster 4-like cells expressed high levels (**Figure 6B**, left). In addition, the mRNA levels of OX40, another activation marker, was expressed in a similar pattern of low expression in cluster 1-like cells and high expression in cluster 4-like cells (**Figure 6B**, right).

We next delved into transcriptomic features pertinent to HIV permissivity and cell death. Cluster 1-like cells preferentially expressed genes for the HIV restriction factors SERINC5, SAMHD1, APOBEC3G, MX2, TRIM32, ISG15 (43–48), which may explain their low permissivity to productive infection (**Figure 6C**). In contrast, cluster 4-like cells expressed lower level of these restriction factor genes, which may explain why these cells allow the virus to complete its life cycle.

Interestingly, cluster 1-like cells highly expressed the genes for caspase 1, caspase 4, and gasdermin D, which are involved in inflammatory pyroptosis. The gene for caspase 3, classically associated with apoptosis but more recently also shown to be linked to pyroptotic death (49, 50), was also upregulated in these cells (**Figure 6D**). The preferential expression of these cell death-associated genes in cluster 1-like cells over cluster 4-like cells suggests that these cells are primed for pyroptotic death (**Table S3**).

In summary, these transcriptomics data demonstrate that the killing of bystander Tm cells is associated with high expression of intracellular restriction factors and cell death factors. In contrast, cells expressing low levels of viral restriction factors and cell death factors were more highly permissive to HIV infection but relatively spared from virus-induced cell death.

## Discussion

HIV induces death of tissue CD4 T cells through two principal mechanisms:1) productive infection of activated cells followed by non-inflammatory apoptosis and 2) abortive infection of nonpermissive cells leading to inflammatory pyroptosis (18). However, little is known about the use of these death pathways within different subsets of tissue memory T cells. In this study, we have used a tissue-based HLAC system combined with extensive phenotyping by CyTOF and single-cell RNA-seq to examine which subsets of CD4 T cells die in the presence of HIV. We find that specific subsets of memory CD4 T cells are highly susceptible to productive viral infection and direct killing. In contrast, other subsets of memory CD4 T cells, along with naïve CD4 T cells, appear refractory to productive infection and die as bystander cells.

One of our most surprising findings is that susceptibility to productive infection and to death do not necessarily go hand in hand. For instance, we identified a Tm subset (cluster 4) that is highly infected but quite resistant to cell death following productive HIV infection. It is not clear why these cells survive so well, although it is intriguing that these cells express high levels of OX40. OX40 is a member of the TNF receptor superfamily and is implicated in protecting the long-term viability of memory T cells, particularly cells underdoing clonal expansion (51). Kuo and colleagues have described how OX40 signaling in HIV-infected cells leads to upregulation of BIRC5 (survivin) (52), a member of the inhibitor of apoptosis (IAP) family and may act by binding to and inhibiting caspase 3 and caspase 7 (53). Furthermore, in mucosal tissues, HIV appears to both preferentially infect cells expressing high BIRC5, and to further upregulate its expression, perhaps to promote survival and dissemination of the infected cells (33). However, BIRC5 mRNA levels were not convincingly upregulated in cluster 4 Tm cells relative to Tm cells (data not shown). It will be of interest to study BIRC5 expression at the protein level in these cells and to test the effects of a BIRC5 inhibitor such as YM155 on their ability to survive HIV-induced abortive infection (54).

Reciprocally, being non-permissive to infection does not protect cells from dying, as previously shown (23). Our analysis shows that the main subset of memory CD4 T cells in this category (cluster 1) display a CD127+CD25- phenotype. The high susceptibility of CD127+CD25- Tm cells to HIV-associated depletion is consistent with *in vivo* observations that CD127+ cells are lost in HIV-infected individuals (55–57). Our studies suggest that this may be primarily due to bystander mechanisms, since cluster 1 cells were rarely infected productively. As CD127 encodes the alpha chain of the IL7 receptor, and is important for T cell homeostasis and survival, depletion of the CD127+ subset could further enhance T cell loss *in vivo* by reducing T cell restoration capacity.

Interestingly, we recently reported that CD127-expressing memory CD4 T cells are resistant to productive infection by HIV (58), consistent with our current study. However, those cells were not preferentially depleted by HIV, but instead became latently infected. One important difference between this prior study and the current one is the tropism of the virus employed: a CCR5-tropic virus was used in the prior study while a CXCR4-tropic virus was used in the current investigation. We chose to work with a CXCR4-tropic virus because this viral type is associated with extensive T cell depletion (59), and is an established system for assessing bystander killing in lymphoid tissue cells (18, 23). A second difference is how the virus is delivered to the target cell. The studies involving R5-tropic virus infection entailed primarily cell-free viral infection whereas the infection in our study is primarily driven by cell-to-cell transmission, which is critically important for effective bystander cell killing (24). Indeed, when cells producing R5-tropic HIV are mixed with CCR5 expressing cells purified from tonsils, pyroptotic bystander cell death is detectable (18).

We observe that preferentially killed cells express especially high levels of CXCR4. These results, coupled with our prior finding that increased levels of viral entry are required for bystander death (24), suggest that the quantity of X4-HIV entering into CD127-expressing CD4 Tm cells shapes the outcome. While the high expressors preferentially undergo bystander cell death, presumably because high CXCR4 levels facilitate transfer of virus from productively infected cells, the low CXCR4 expressors are potentially more prone to undergo productive infection.

Besides CXCR4, other cellular factors also seem to affect cell fate after HIV entry. For example, the CD127+CD25- Tm cells (similar to cluster 1 cells preferentially undergoing bystander cell death) exhibit an overall resting-like phenotype defined by low expression of activation markers. In addition, these cells are distinguished by increased expression of several HIV restriction factors, which further reinforces their nonpermissive state, encouraging abortive infection and caspase-1 dependent pyroptosis (18, 23). Furthermore, these cells appear primed for bystander cell death as evidenced by increased expression of the genes for caspase-1, caspase-4 and gasdermin D. Each of these proteins are important effectors in the pyroptotic pathway, and their synchronized upregulation may promote this rapid death mechanism.

The features we describe of Tm subsets preferentially prone to undergo productive infection vs. abortive cell death likely will not hold true in blood. Unstimulated blood CD4 T cells are highly resistant to abortive infection and pyroptosis unless first cultured with lymphoid tissue cells under conditions where cell-to-cell interactions occur (17). These findings underscore the importance of studying HIV pathogenesis in cells from lymphoid tissues rather than from PBMCs, because at least some biological responses are strikingly different.

Of note, the cluster 1 cells also expressed caspase-3 mRNA. Caspase 3 is generally regarded as an important inducer of apoptosis rather than pyroptosis. However, recent studies suggest cross-talk between the apoptotic and pyroptotic pathways of programmed cell death. For example, activation of caspase-3 by chemotherapeutic drugs can stimulate pyroptosis. In this case, caspase-3 cleaves gasdermin E, liberating an active N-terminal fragment that promotes membrane pore formation (49). In another example of caspase cross-talk, activated caspase 1 can activate caspase 3 and 7, inducing an apoptotic form of cell death as a safeguard to ensure the death of cells that initiate but do not complete pyroptotic pathway of programmed cell death (50).

In summary, our studies highlight how the response to HIV infection can sharply differ within different subsets of CD4 memory T cells. Some cell subsets readily undergo productive infection and survive, while others die after productive infection. Other subsets mainly die as bystanders as a consequence of abortive infection and pyroptotic cell death. Bystander death is favored when infection involves X4-tropic viruses, under such conditions is associated with higher levels of expression of CXCR4. Additionally, susceptible bystander cells appear primed for death by pyroptosis based on increased mRNA expression of the inflammatory caspases (caspase 1 and caspase 4) as well as the pyroptotic executioner, gasdermin D. The nature of the signals underlying this priming remains unclear but likely results from the unique environment provided by lymphoid tissues. Effective interdiction of such signaling could prevent both bystander cell death and the inflammation it engenders.

## Materials and Methods

### Cells and Media Supplements

Human tonsils were obtained from the Cooperative Human Tissue Network (CHTN) during routine tonsillectomies, mainly for sleep disorders. These tissues were processed as previously described (18). Briefly, HLAC single-cell suspensions were created by dissection and then by pressing the Tonsil tissue through a 40-µm mesh. Live lymphocytes were then isolated from the single cell suspensions by Ficoll density gradient centrifugation. HLAC cells were cultured in tonsil culture media that consisted of RPMI 1640 supplemented with 15% fetal bovine serum (FBS) (Corning), 100 mg/mL gentamicin (Gibco), 200 mg/mL ampicillin (Sigma-Aldrich), 1 mM sodium pyruvate (Sigma-Aldrich), 1% non-essential amino acids (Mediatech), 1% Glutamax (ThermoFisher), and 1% Fungizone (Invitrogen).

### Antibodies and Reagents

Details about the antibodies used in flow cytometry staining are listed in supplemental table 1 and for CyTOF in supplemental table 2. All CyTOF antibodies were purchased from Fluidigm. HLADR Qdot(112Cd) was purchased pre-conjugated. All other CyTOF antibodies were purchased unconjugated and then conjugated with elemental isotopes in house using MAXPAR^®^ X8 Ab Label Kits from Fluidigm.

### Virus Preparation

The pNLENG1-IRES-GFP clone was derived from NL4-3 as previously described (59). The pro-viral expression vector DNA encoding the pNLENG1-IRES-GFP reporter virus was transfected into 293T cells using the Promega Fugene HD transfection reagent (catalog no. E2311) and cultured at 37°C. Media was replaced after 16 hours. Culture supernatants were collected 24 and 48 hours post-transfection. Virions were concentrated by ultracentrifugation at 20,000 rpm (32198 g) for 2 hours on a Beckman Coulter Optima XE-90. Gag-p24 levels of the HIV.GFP viral stocks were quantitated by enzyme-linked immunosorbent assay (ELISA) using the Lenti-X™ p24 Rapid Titer Kit from Clontech (catalog no. 632200).

### HLAC Killing Assay by Spinoculation

One million HLAC cells were mixed with HIV-1.GFP (NLENG1-IRES-GFP) or mock control (culture media) in 100 µl total volume and cultured in V-bottom wells of a 96-well plate. To achieve different levels of T cell depletion, we applied 3 different HIV amounts (25, 50, and 100 ng p24 gag of HIV) and treated them as technical repeats in the current study. Cells were centrifuged at 25°C at 1200 x g for 2 hours and then cultured at 37°C as a pellet (23). Both infected and uninfected (mock) HLAC cells were collected and processed for FACS or CyTOF staining followed by calculation of % live and % infection as described in the following sections. A total of 6 donors were included in the current study and technical repeats from each donor with more than of 10% CD4 T cell depletion were analyzed.

### FACS Analysis and Gating Strategy

Cells collected from HLAC cultures were processed for fluorescence-activated cell sorting (FACS) staining using a live-dead cell discriminator dye (Zombie Aqua) and stained with fluorescently labeled antibodies specific for the cell markers in supplemental table 1. Data were collected on a BD LSRFortessa™ X-20 Cell Analyzer flow cytometer and analyzed with FlowJo software from BD bioscience. At least 100,000 events per sample were collected, and at least 50,000 live cells were included in the subsequent data analysis. Gating strategies for each cell subset are shown in **Supplementary Figure S1** (Cells, Singlet, Live, CD8 T, CD4 T, Tn, Tm, Tem, Tcm, and Tfh).

### CyTOF Staining and Data Processing

CyTOF staining was conducted as previously described (32). Briefly, cells collected from HLAC cultures were first stained for cell surface markers using antibodies conjugated with elemental isotopes listed in supplemental table 2. For live-dead staining, cells were treated with 139In-loaded-maleimide-DOTA (Macrocyclics) at 5 µg/ml on ice for 30 minutes, and cisplatin (Sigma-Aldrich) at 25 µM at room temperature for 60 seconds. Cells were then immediately fixed with 2% paraformaldehyde overnight at 4°C. Cells from multiple samples were barcoded using a Cell-ID™ 20-Plex Pd Barcoding Kit from Fluidigm, and combined before being permeabilized using the eBioscience™ Foxp3/Transcription Factor Staining Buffer Set. Cells were then stained for intracellular antigens including GFP, Gag, Foxp3, cleaved caspase-3, CTLA4, SAMHD1, and IFI16. CyTOF data were collected from a Fluidigm CyTOF2 Helios Mass Cytometer, normalized, and de-barcoded by CyTOF^®^ Software from Fluidigm. Expression data for all parameters in each cell were next analyzed in the form of Flow Cytometry Standard (FCS) files in FlowJo™ software from BD bioscience. Data were pre-gated on Cells, Intact cells, Singlets, and Live cells before further analysis (**Figure S2**). A range of 10,000-130,000 cells (Live singlets post-CD8 normalization) per sample were included in the CyTOF analyses. tSNE and FlowSOM analysis were conducted in the Cytobank platform from Cytobank, Inc.

### PP-SLIDE Analysis

PP-SLIDE was previously introduced and validated as an approach to trace HIV-remodeled cells back to their original pre-infected state (31–34). The detailed description and R script were made available in our previous publication [Neidleman et al. eLife 2020 (32)]. In the current study, we implemented PP-SLIDE to predict the pre-infection cell marker expression of all cells in the HIV-exposed HLAC culture. As an example of the single-cell analysis using PP-SLIDE, for Infe-cell #1, we calculated the Euclidian distances between Infe-cell #1 and each cell from the uninfected culture. The cell in the uninfected culture found to have the shortest Euclidian distance with Infe-cell #1 is identified as the predicted precursor of Infe-cell #1 (kNN_Infe-cell #1_). The cell marker expression of the kNN cells was used for phenotyping the infected and bystander cells in all our CyTOF data.

### Measuring the Level of HIV-Productive Infection

To evaluate the level of productive infection in specific cell subsets, we first gated on the subset of interest in FlowJo and then measured the cell number in this subset in uninfected and infected samples. The level of HIV-productive infection in a selected subset, for example, subset S1, is calculated by the equation below:

*% infection (S1): percent of productively infected cells in subset S1*

*% infection (S1) = number of S1 cells in infected sample/number of S*1 *cells in uninfected sample x 100*

Similarly, the level of productive HIV infection in the Tm population is calculated as: number of productively infected cells in the infected sample/number of Tm cells in the uninfected sample.

Tm subsets with a higher proportion of productively infected cells (% infection) than total Tm cells are referred to as “preferentially-infected subsets”.

### Measuring the Level of HIV-Associated Killing

To evaluate the level of HIV-associated killing in specific cell subsets, we first gated on the subset of interest in FlowJo and then measured the following cell numbers (for example, subset S1):

*cNum_UnS1_: Cell number of S1 subset from uninfected sample*

*cNum_InfeS1_: Cell number of S1 subset from infected sample*

*cNum_UnCD8_: Cell number of CD8 T cells from uninfected sample*

*cNum_InfeCD8_: Cell number of CD8 T cells from infected sample*

The level of HIV-associated killing is indicated by % killing and is calculated using the following equation:

*% killing (S1): percent of HIV-associated killing of cells in subsets of interest S1 compared to uninfected sample and normalized with CD8 T cell number.*

*% killing (S1) = (cNum_InfeS1_ ÷ cNum_UnS1_) X (cNum_UnCD8_ ÷ cNum_InfeCD8_) X100*

Tm subsets with a higher level of HIV-associated killing than that of total Tm cells are defined as “preferentially-killed subsets”.

### Single-cell RNA sequencing

T cells were isolated from HLAC using Immunomagnetic negative selection (StemCell Technologies), and dead cells were removed using a dead cells removal kit (Miltenyi Biotec). The cells were stained with a panel of TotalSeq-A human antibodies (BioLegend) according to manufacturer’s protocol. Stained cells were then loaded onto a Chromium Next GEM chip G and both ADT (Antibody Derived Tag) and GEX (Gene Expression) libraries were generated using Chromium Next GEM Single Cell 3**’ **Reagent Kits v3.1 (10X genomics) for next generation sequencing. A range of 11,000-13,000 events per sample were collected for the subsequent single-cell RNAseq analysis. The data was preprocessed in Cell Ranger for alignment against the human genome and further analyzed in SeqGeq ^®^.

## Data Availability Statement

The datasets presented in this study can be found in online repositories. The names of the repository/repositories and accession number(s) can be found below: https://datadryad.org/stash, https://datadryad.org/stash/share/rGPu0kf279Om-HMWZmFnL-elCzILgnb2KVfppH3McxU.

## Author Contributions

All authors listed have made a substantial, direct, and intellectual contribution to the work and approved it for publication.

## Funding

This research was supported by the National Institutes of Health (R01 DA044605, P01 10018714, P01 AI124912, P30 DK063720, R01 AI127219, R01 AI147777, P01 AI131374, UM1 AI164559, S10 OD018040). We also gratefully acknowledge funding support from the James B. Pendleton Charitable Trust. This publication was made possible with help from the UCSF-Gladstone Center for AIDS Research (CFAR), an NIH-funded program (P30 AI027763). We also acknowledge the funding from the HOPE Collaboratory (UM1 AI164559).

## Conflict of Interest

The authors declare that the research was conducted in the absence of any commercial or financial relationships that could be construed as a potential conflict of interest.

## Publisher’s Note

All claims expressed in this article are solely those of the authors and do not necessarily represent those of their affiliated organizations, or those of the publisher, the editors and the reviewers. Any product that may be evaluated in this article, or claim that may be made by its manufacturer, is not guaranteed or endorsed by the publisher.
