# Supplementary material for: Subsets of Tissue CD4 T Cells Display Different Susceptibilities to HIV Infection and Death: Analysis by CyTOF and Single Cell RNA-seq
Source: Front Immunol. 2022 Jun 16;13:883420. doi: 10.3389/fimmu.2022.883420 (PMC9245423; doi:10.3389/fimmu.2022.883420)
Supplement: Supplementary file 1 [file DataSheet_1.pdf]

Supplemental table 1. Antibodies and reagents used for Flow Cytometry

Fluorescence	Cell Markers	Vendor	Catalog no.	Clone
PerCP-Cy™5.5	CD3	BD Biosciences	340948	Clone SK7 (ASR)
PE-Texas Red®	CD4	Thermo Fisher	MHCD0417	clone S3.5
Qdot 655	CD45RA	Thermo Fisher	MEM-56	Q10069
Brilliant Violet 421™	CCR7	Biolegend	353207	G043H7
1)Biotin-CXCR5; 2)Strep-QD605	CXCR5	1)R&D systems; 2)Thermo Fisher	1)FAB190B; 2)Q10101MP	51505
APC	PD1	Biolegend	329908	EH12.2H7
PECy5	CD8	BD Biosciences	561951	RPA-T8 (RUO)
GFP	HIV	-	-	-
Zombie Aqua (Amcyan)	Live vs Dead	Biolegend	423102	-

Supplemental table 2. **Antibodies and reagents used for CyTOF**

Elemental Isotope	Cell Markers	Vendor	Catalog no.	Clone
102-110Pd	Barcodes	Fluidigm	201060	-
139La	CD30	Biolegend	333902	BY88
141Pr	CD19	Biolegend	302247	HIB19
142Nd	Caspase 3 cleaved	Fluidigm	3142004A	D3E9
143Nd	CD45RA	Fluidigm	3143006B	HI100
144Nd	CCR5	Fluidigm	3144007A	NP6G4
145Nd	GFP	Biolegend	338002	FM264G
146Nd	IFI16	Santa Cluz	sc-8023	1G7
147Sm	CD7	Fluidigm	3147006B	CD76B7
148Nd	ICOS	Fluidigm	3148019B	C398.4A
149Sm	CCR4	Fluidigm	3149003A	205410
150Nd	CD32	BD	557333	FLI8.26
151Eu	CD2	Fluidigm	3151003B	TS1/8
152Sm	CD45RO	Biolegend	304239	UCHL1
153Eu	CD62L	Fluidigm	3153004B	DREG56
154Sm	SAMHD1	Bethyl Laboratories	A303-691A	Ag3287
155Gd	CCR6	Biolegend	353427	G034E3
156Gd	CD29	Fluidigm	3156007B	TS2/16
157Gd	CCR2	Biolegend	357202	K036C2
158Gd	OX40	Fluidigm	3158012B	ACT35
159Tb	CCR7	Fluidigm	3159003A	G043H7
160Gd	CD28	Fluidigm	3160003B	CD28.2
161Dy	CTLA-4	Fluidigm	3161004B	14D3
162Dy	CD69	Fluidigm	3162001B	FN50
163Dy	CXCR3	Fluidigm	3163004B	G025H7
164Dy	CD95/FasL	Fluidigm	3164008B	DX2
165Ho	CD127	Fluidigm	3165008B	A019D5
166Er	CXCR5	Biolegend	356902	J252D4
167Er	CD27	Fluidigm	3167006B	L128
168Er	CD8	Fluidigm	3168002B	sk1
169Tm	CD25	Fluidigm	3169003B	2A3
170Er	CD3	Fluidigm	3170001B	UCHT1
171Yb	PD1	Biolegend	329941	EH12.2H7
172Yb	CD38	Fluidigm	3172007B	HIT2
173Yb	Foxp3	Biolegend	320102	206D
174Yb	CD4	Fluidigm	3174004B	SK3
175Lu	CXCR4	Fluidigm	3175001B	12G5
176Yb	CD57	Biolegend	322325	HNK-1
Qdot (112Cd)	HLADR	Life Technologies	Q22158	Tu36
209Bi	Gag (KC57)	Beckman Coulter	IMBULK1	FH190-1-1

Supplemental table 3. Summary of cluster 1 to 4

	Productively infected	HIV induced killing	Dominant Killing Mechanism	Key Markers
**Cluster 1**	No	Preferentially Killed	Bystander killing by pyroptosis	CD127+ CD25-
**Cluster 2**	Yes	Preferentially Killed	Both	CXCR5+ PD1+ CD25- CD127- CD57-
**Cluster 3**	Yes	Preferentially Killed	Both	CD57+ PD1+
**Cluster 4**	Yes	Killing < Average Tm	Productive infection-induced killing	CD25+ PD1+ CD57-

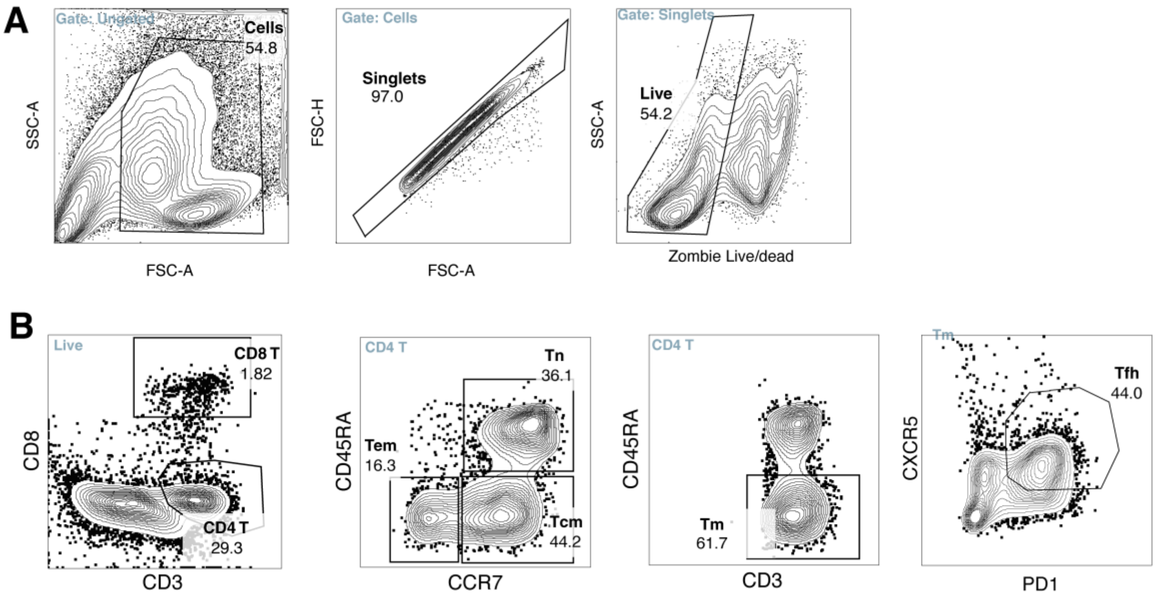

Supplemental Figure S1. Flow cytometry (FACS) gating strategy.

A. Example of sequential FACS gates: Cells (exclusion of cell debris), Singlets (removal of doublets), Live (exclusion of dead cells). **B.** FACS gating strategies for T cell subsets: CD8 T (CD3+CD8+), CD4 T (CD3+CD8-), Tn (CD3+CD8-CD45RA+CCR7+), Tem (CD3+CD8-CD45RA-CCR7-), Tcm (CD3+CD8-CD45RA-CCR7+), Tm (CD3+CD8-CD45RA-), and Tfh cells (CD3+CD8-CD45RA-CXCR5+PD1+). Preceding parent gates are indicated at the upper left corner. Numbers correspond to percentages of cells in each gate.

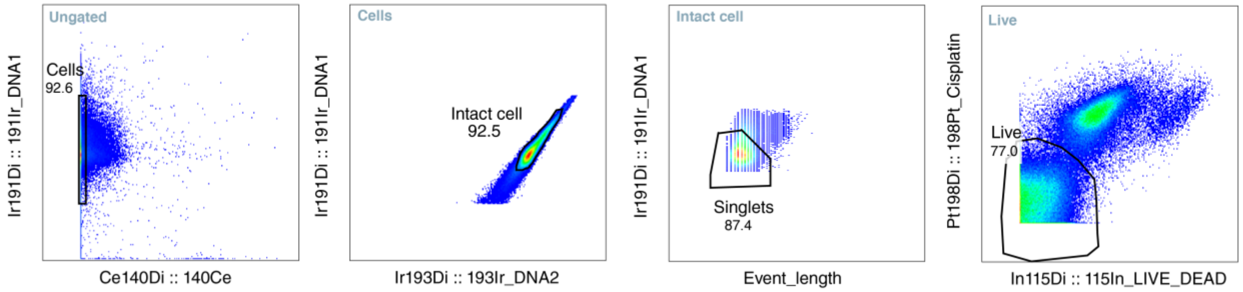

Supplemental Figure S2. CyTOF gating strategy.
Example of initial sequential gates: Cells (exclusion of beads), Intact cells (exclusion of debris), Singlets (removal of doublets), Live (exclusion of dead cells). Numbers correspond to percentages of cells in each gate. Preceding parent gates are indicated at the upper left corner. Numbers correspond to percentages for each gate.

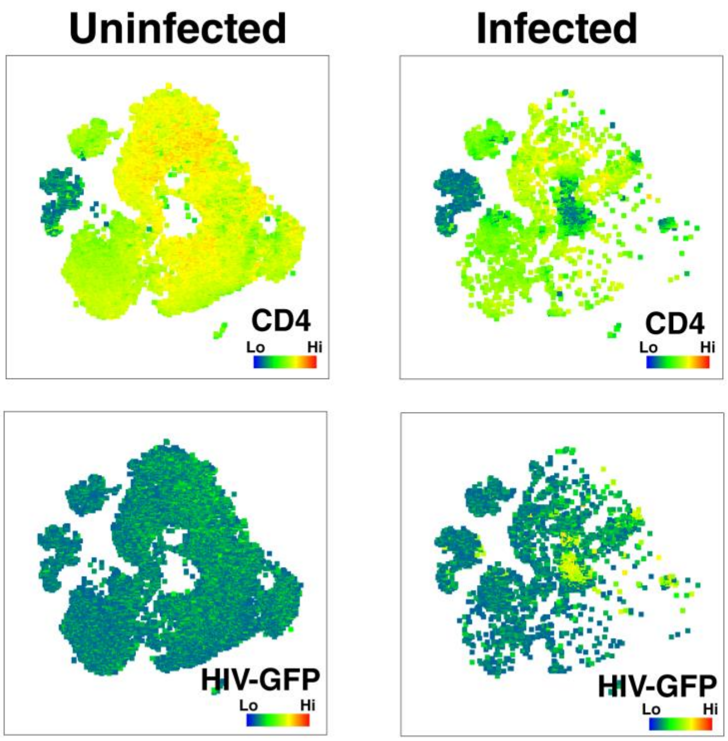

Supplemental Figure S3. CD4 and HIV-GFP expression in uninfected and HIV infected CD4 T cells

HLAC cells from uninfected and infected cultures were pre-gated on CD4 T cells (CD3+CD8-). The expression of CD4 (Top panels) and HIV-GFP (Lower panels) are shown in tSNE heatmaps.

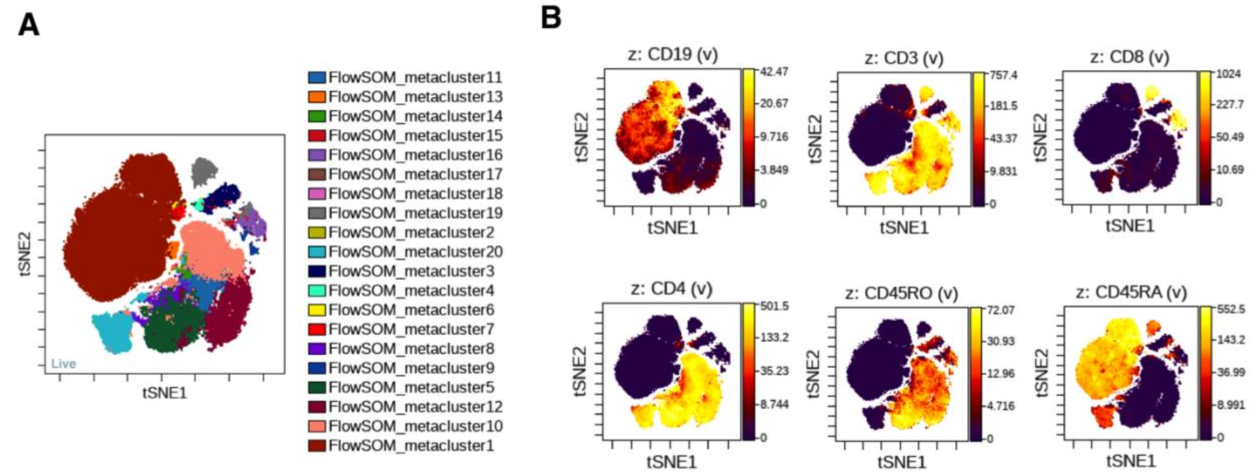

Supplemental Figure S4. Identification of CD8 T, CD4 T, Tm and Tn cells by FlowSOM

A. Total HLAC cells were divided into 20 FlowSOM clusters (F1-F20) and overlayed in a tSNE plot with different colors. **B.** tSNE heatmaps of the expression of selected cell markers (arcsinh-transformed) for classifying the FlowSOM clusters corresponding to each main subset including B (CD19+), CD8 T (CD3+CD8+CD4-), Tm (CD4+CD45RO+CD45RA-), and Tn (CD4+CD45RO-CD45RA+) cells.

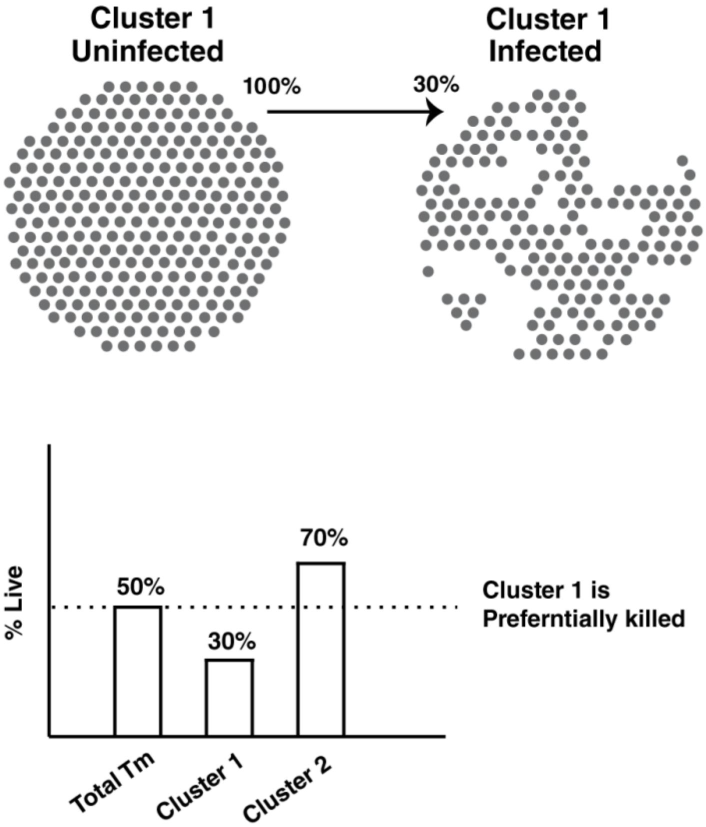

Supplemental Figure S5. Schematic of process to define preferentially killed subsets

Upper panel: Schematic of cell loss in a FlowSOM cluster (cluster 1) from 100% (uninfected culture) to 30% (infected culture) after HIV infection. Lower panel: Schematic showing that the cell loss in cluster 1 was higher than the cell loss of total Tm cells. Cluster 1 was defined as a preferentially killed cluster.

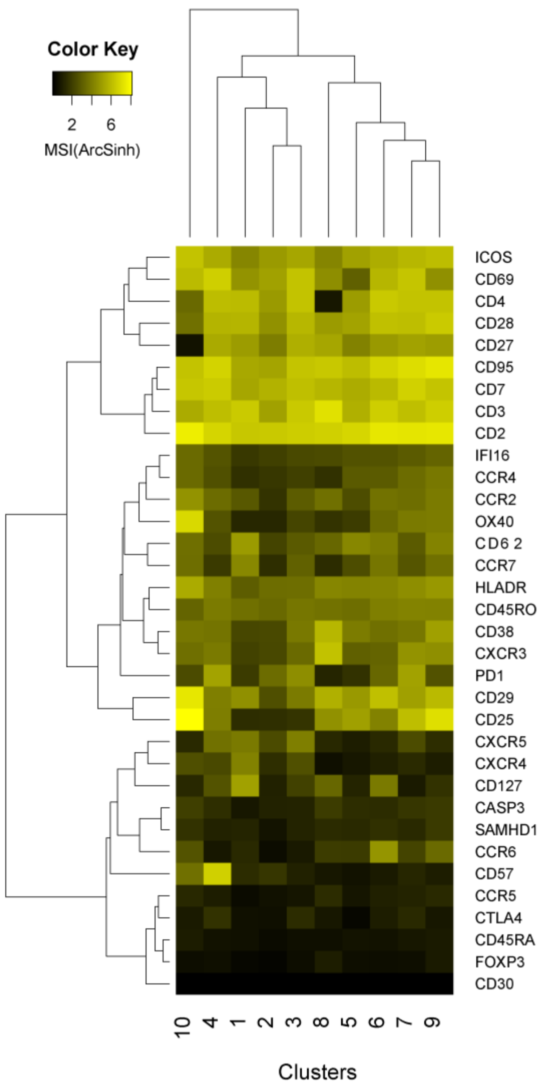

Supplemental Figure S6. A hierarchically clustered heatmap showing cell marker expression profile of 10 Tm FlowSOM clusters

Yellow and black colors represent the low- and high- expression of 34 Tm markers measured by CyTOF. All MSI values were arcsinh transformed.

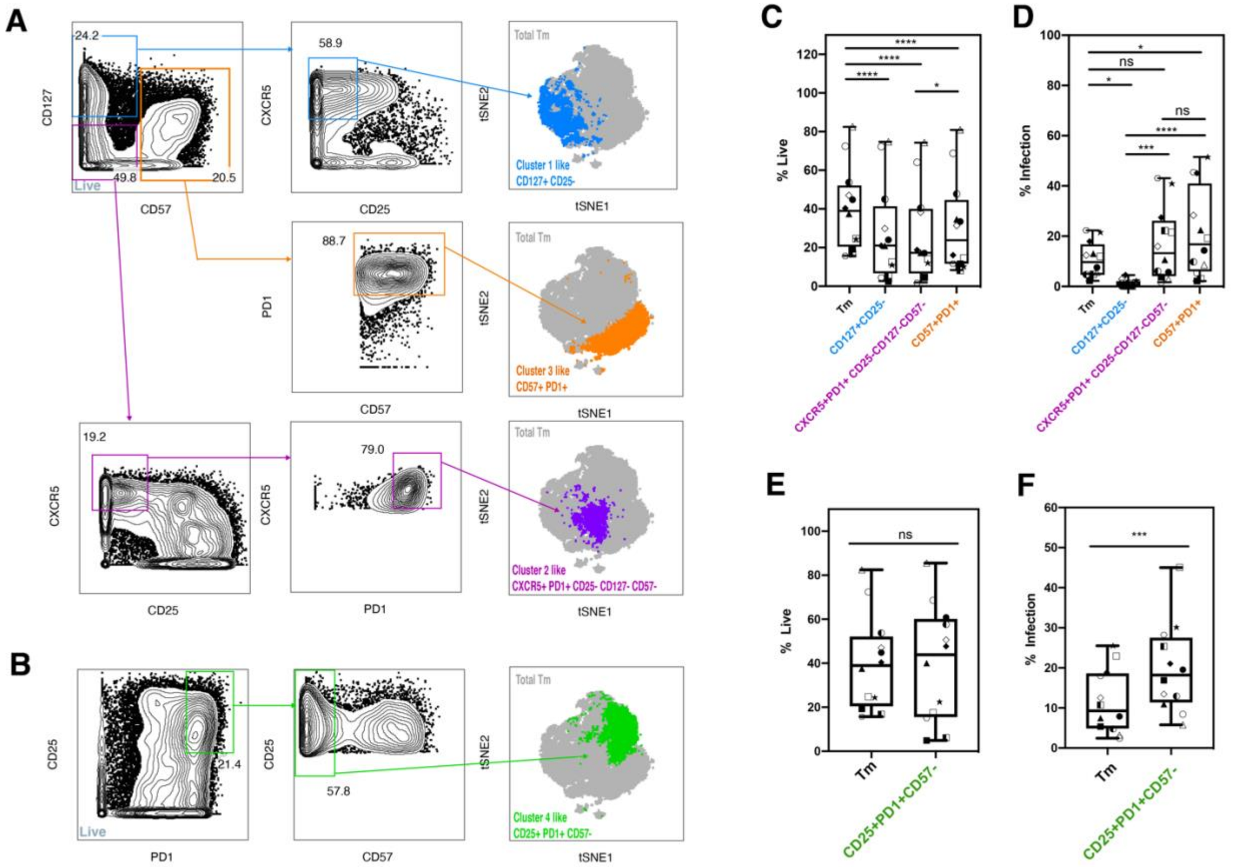

Supplemental Figure S7. Re-identification of preferentially-killed and -infected Tm subsets (FlowSOM clusters 1-4) by manual gating strategies.

Manual gating strategies for re-identifying the FlowSOM Tm subsets with less than 5 key parameters, as listed in figure 5E. These subsets were: **A.** cluster 1 (blue), cluster 2 (purple), cluster 3 (orange), and **B.** cluster 4 (green). *A & B right panels*: subsets generated by manual gating (Cluster 1-4 like) were overlayed with total Tm (gray) in tSNE plots, showing that the re-identified subsets and the clusters defined by FlowSOM in figure 4 occupied a similar tSNE locations. Preceding parent gates are indicated at the upper left corner. Numbers correspond to percentage of cells in each gate. **C.** % live and **D.** % infection of manual gating subsets (cluster 1-3 like) plotted as box plots. The plots represent data from 6 donors. Each donor is represented as a shape and technical repeats with different type of fill (details described in materials and methods). **p≤0.05; ****p≤0.0001; no label: not significant, p>0.05.* Significance was measured by one-way ANOVA with repeated measurements followed by post-hoc tests of multiple comparisons correction. (n = total paired-wise comparisons). **E.** % live and **F.** % infection of manual gating subset (cluster 4 like) plotted as box plots. The plots represent data from 6 donors. Each donor is represented as a shape and technical repeats with different type of fill. ****p≤0.001; n.s., not significant, p>0.05*. Significance was measured by paired Student's T test.

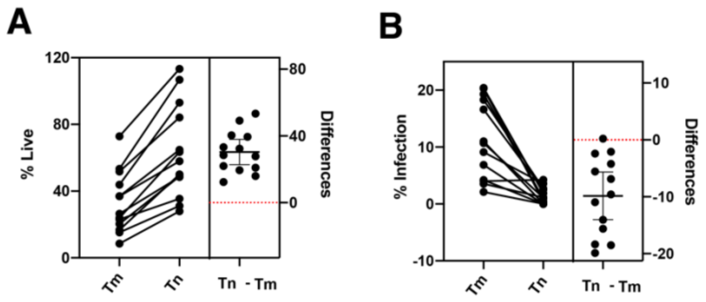

Supplemental Figure S8. Differences in killing and infection level between Tm and Tn cells paired by donor and experiment.

In figure 3B, we found significant differences between Tm and Tn cells in their susceptibility to killing (% live) and to infection (% infection) by HIV. To show the differences and their magnitude between Tm and Tn cells from the same infected culture, here we show the estimation plots generated by GraphPad Prism following paired Student's T tests. Each dot represents one donor and one experiment. For each panel, the left graph connects Tm and Tn data from the culture (same donor, same experimental technical replica), and the right graphs show the differences in % live or % infection values between Tn and Tm cells from the same infected culture. In the right graphs, the error bars show the mean and 95% confidence interval. The red dotted line marks y=0. In all experiments, Tn cells were more likely than Tm cells to survive (y>0), and in most experiments, Tm cells were more susceptible than Tn cells to productive infection (y<0).

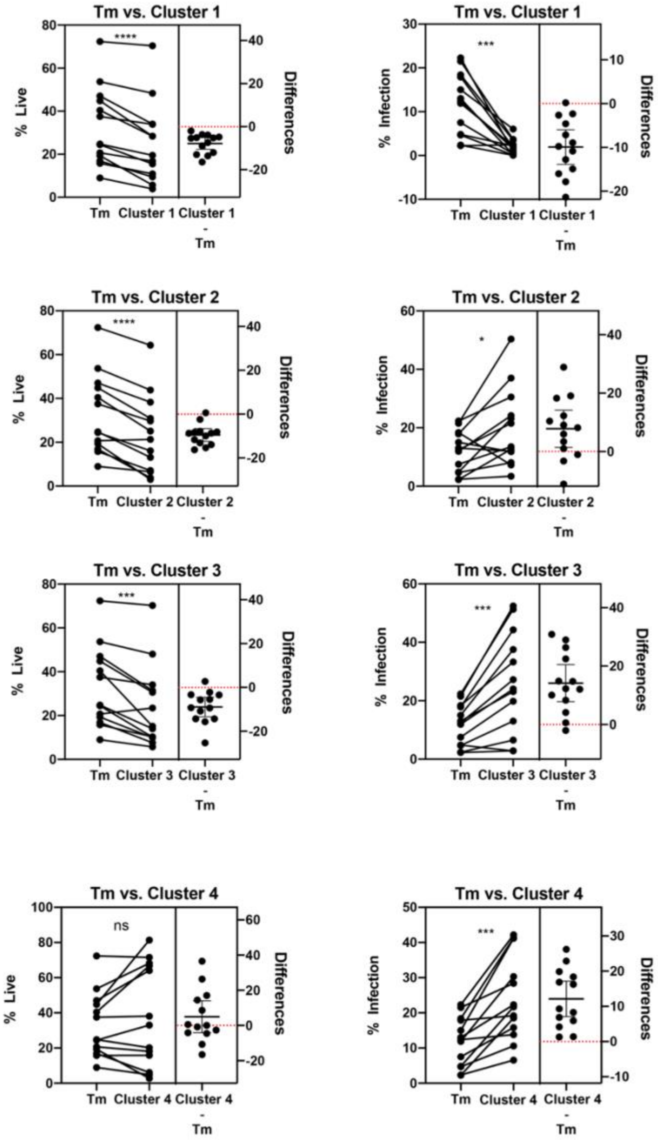

Supplemental Figure S9. Estimation plots showing the paired difference of killing and infection level between Tm and Tm subsets (cluster 1-4).

In figure 4B, we found that clusters 1-3 were preferentially killed with a significantly higher level of killing and clusters 2-4 were preferentially infected with a significantly higher level of infection. To show the paired effect and the magnitude of differences between Tm and each of the 4 clusters from the same infected culture, here we show the estimation plots generated by GraphPad Prism following paired Student's T tests. In detail, the left y-axes data (% live or % infection) from the same infected culture were connected with a solid line to show the paired effect. The right y-axes data showed the difference of % live or % infection between Tm and each cluster from the same infected culture, calculated by cluster minus Tm, and presented with mean with 95% confidence interval. The right y-axes value =0 indicated no difference and was labeled with red dotted lines. The right y-axes value >0 indicated a positive difference and <0 indicated a negative difference.
